# Daily Chlorhexidine Bath for Health Care Associated Infection Prevention (CLEAN-IT): protocol for a multicenter cluster randomized crossover open-label trial

**DOI:** 10.62675/2965-2774.20240053-en

**Published:** 2024-09-18

**Authors:** Bruno Martins Tomazini, Thabata Silva Veiga, Renato Hideo Nakagawa Santos, Viviane Bezerra Campos, Samira Martins Tokunaga, Elton Sousa Santos, Leticia Galvão Barbante, Renato da Costa Maia, Karina Leal Negrelli, Nanci Valeis, Eliana Vieira Santucci, Ligia Nasi Laranjeira, Fernando Azevedo Medrado, Thiago Costa Lisboa, Bruno Adler Maccagnan Pinheiro Besen, Antônio Paulo Nassar, Viviane Cordeiro Veiga, Adriano José Pereira, Alexandre Biasi Cavalcanti

**Affiliations:** 1 HCor Research Institute HCor-Hospital do Coração São Paulo SP Brazil HCor Research Institute, HCor-Hospital do Coração - São Paulo (SP), Brazil.; 2 Brazilian Research in Intensive Care Network São Paulo SP Brazil Brazilian Research in Intensive Care Network (BRICNet) – São Paulo (SP), Brazil.; 3 Hospital Sírio-Libanês São Paulo SP Brazil Hospital Sírio-Libanês - São Paulo (SP), Brazil.; 4 Hospital de Clínicas de Porto Alegre Universidade Federal do Rio Grande do Sul Porto Alegre RS Brazil Hospital de Clínicas de Porto Alegre, Universidade Federal do Rio Grande do Sul -Porto Alegre (RS), Brazil.; 5 Hospital das Clínicas Faculdade de Medicina Universidade de São Paulo São Paulo SP Brazil Medical Intensive Care Unit, Hospital das Clínicas, Faculdade de Medicina, Universidade de São Paulo - São Paulo (SP), Brazil.; 6 Hospital Israelita Albert Einstein São Paulo SP Brazil Hospital Israelita Albert Einstein - São Paulo (SP), Brazil.; 7 AC Camargo Cancer Center São Paulo SP Brazil AC Camargo Cancer Center - São Paulo (SP), Brazil.; 8 BP - A Beneficência Portuguesa de São Paulo São Paulo SP Brazil BP - A Beneficência Portuguesa de São Paulo - São Paulo (SP), Brazil.

**Keywords:** Cross infections, Chlorhexidine, Anti-infective agents, local, Baths, Soaps, Critical illness, Nosocomial infections, Sepsis, Length of stay, Intensive care units

## Abstract

**Background:**

Critically ill patients are at increased risk of health care-associated infections due to various devices (central line-associated bloodstream infection, catheter-associated urinary tract infection, and ventilator-associated pneumonia), which pose a significant threat to this population. Among several strategies, daily bathing with chlorhexidine digluconate, a water-soluble antiseptic, has been studied as an intervention to decrease the incidence of health care-associated infections in the intensive care unit; however, its ability to reduce all health care-associated infections due to various devices is unclear. We designed the Daily Chlorhexidine Bath for Health Care Associated Infection Prevention (CLEAN-IT) trial to assess whether daily chlorhexidine digluconate bathing reduces the incidence of health care-associated infections in critically ill patients compared with soap and water bathing.

**Methods:**

The CLEAN-IT trial is a multicenter, open-label, cluster randomized crossover clinical trial. All adult patients admitted to the participating intensive care units will be included in the trial. Each cluster (intensive care unit) will be randomized to perform either initial chlorhexidine digluconate bathing or soap and water bathing with crossover for a period of 3 to 6 months, depending on the time of each center’s entrance to the study, with a 1-month washout period between chlorhexidine digluconate bathing and soap and water bathing transitions. The primary outcome is the incidence of health care-associated infections due to devices. The secondary outcomes are the incidence of each specific health care-associated infection, rates of microbiological cultures positive for multidrug-resistant pathogens, antibiotic use, intensive care unit and hospital length of stay, and intensive care unit and hospital mortality.

**Conclusion:**

The CLEAN-IT trial will be used to study feasible and affordable interventions that might reduce the health care-associated infection burden in critically ill patients.

## INTRODUCTION

Health care-associated infections (HCAIs) are common preventable illnesses in hospitalized critically ill patients^( 1 )^ and are associated with increased health care costs, length of stay, and mortality.^( 2 - 5 )^ Critically ill patients are at increased risk for HCAIs due to a myriad of factors, such as the use of invasive devices (endotracheal tubes, central lines, and urinary catheters) and their underlying critical illness. Practices such as care, device insertion bundles, and hand hygiene are the cornerstones of infection prevention^( 6 - 9 )^ and a major focus of infection and prevention control services, with several clinical trials evaluating infection prevention strategies in the critical care setting.^( 10 - 15 )^

Chlorhexidine digluconate (CHG) is a water-soluble, low-foaming, topical antiseptic agent included on the list of essential medicines of the World Health Organization;^( 16 )^ CHG exhibits activity against gram-positive and gram-negative bacteria, yeasts, and lipophilic viruses with a possible benefit of residual antibacterial activity.^( 17 )^ Chlorhexidine digluconate has been used in impregnated vascular catheter dressings, skin preparation for invasive procedures, preoperative bathing, and oral decontamination.^( 18 - 20 )^

The use of daily bathing with CHG to reduce HCAIs in critically ill patients has been evaluated in several studies, with mixed results indicating that it decreases the risk of some infections (central line-associated bloodstream infection [CLABSI] and bacteremia) while increasing the risk of others (ventilator-associated pneumonia [VAP]).^( 14 , 15 , 21 , 22 )^ The role of CHG bathing in reducing HCAIs has not been properly evaluated in low- and middle-income countries (LMICs), where the incidence and burden of HCAIs are greater.^( 1 , 23 , 24 )^ A cluster randomized design is desirable when studying interventions to reduce HCAIs given the possible benefit of the spillover effect and the reduction of contamination between the treatment and control groups.^( 25 , 26 )^

The Daily Chlorhexidine Bath for Health Care Associated Infection Prevention (CLEAN-IT) trial is a cluster randomized crossover trial to evaluate the use of daily chlorhexidine bathing in critically ill patients in reducing device-associated HCAIs (CLABSI, catheter-associated urinary tract infection [CAUTI], and VAP) compared with soap and water bathing.

## METHODS

The Standard Protocol Items: Recommendations for Interventional Trials (SPIRIT) guidelines were followed for this report.^( 27 )^ The final trial report will follow the Consolidated Standards of Reporting Trials (CONSORT) statement and its extension to cluster randomized trials.^( 28 )^ The trial was registered with ClinicalTrials.gov (NCT05485051) before the inclusion of the first patient.

### Design

The CLEAN-IT trial is an investigator-initiated, multicenter, open-label, cluster-randomized crossover clinical trial assessing the effectiveness of daily chlorhexidine baths in reducing the incidence of device-associated HCAIs in critically ill patients compared with soap and water baths.

The study was conducted in 23 Brazilian intensive care units (ICUs) and is nested in the IMPACTO-MR platform,^( 29 )^ a research platform that collects prospective observational data from more than 50 Brazilian ICUs.

**Figure 1:**
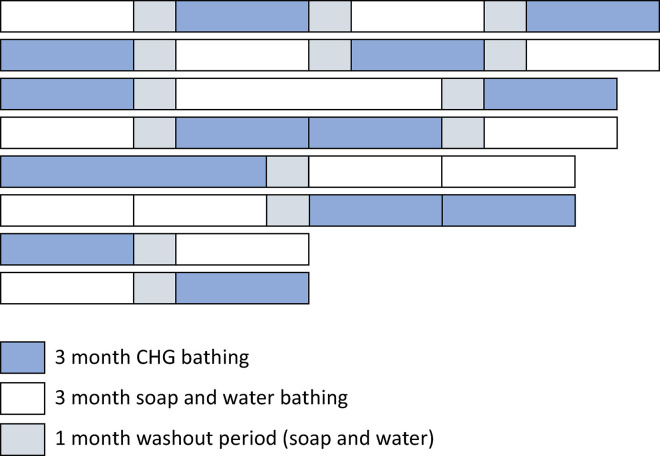
Schematic of possible allocation sequences.

Each participating ICU is a cluster that was randomized to perform the interventions (CHG bathing or soap and water bathing) with crossover for a period of 3 to 6 months, depending on the time of each center’s entrance to the study, with a 1-month washout period between CHG bathing and soap and water bathing transitions. Figure 1 shows all possible allocation sequences for the participating ICUs.

CHG - chlorhexidine digluconate.

### Study population and interventions

All adult patients ≥ 18 years of age admitted to the participating ICUs were included in the study. Patients were excluded if they were known to have CHG allergies.

### Chlorhexidine digluconate bath

All patients in the ICU during the CHG bath period who did not meet the exclusion criteria were bathed daily with 2% chlorhexidine digluconate (2% CHG). All participating centers received 2% CHG and gloves (soft polyester gloves specifically for hospital bathing) for all CHG bath periods.

The bathing procedure consisted of applying 2% CHG (any form of dilution of the 2% CHG solution was not allowed) with the provided gloves and using it to bathe the patient, ensuring that all the applicable bathing surfaces were covered with 2% CHG and that there were no areas with visible soiling on the patient. The applicable bathing surfaces included all the body surfaces except the eyes, inner ear, mouth, and areas with loss of skin continuity (e.g., open wounds and burnt regions). Bathing was performed in the craniocaudal direction (head to toe), with the last area bathed being the genital region, which was mandatorily washed with a new pair of gloves. Patients were rinsed according to the usual practices of each center.

In cases of extensive soiling, cleaning of the soiled area could be performed before 2% CHG bathing. Other bath-related aspects (e.g., time of day, number of staff involved in each bath, and bedside or shower bath) and infection control practices and bundles followed each center’s usual practices.

### Soap and water bath

All patients in the ICU during the soap and water bath period and washout periods were bathed daily following each center’s usual practices. Chlorhexidine for bathing at any concentration was forbidden during these periods. The only exception was preoperatory bathing for surgical patients, which was allowed in centers with this practice. Other bath-related aspects (e.g., time of day, number of staff involved in each bath, and bedside or shower bath) and infection control practices and bundles followed each center’s usual practices.

### Washout period

The washout period was defined as 1 month between distinct intervention periods (CHG bath period and soap and water bath period). During this period, all centers used soap and water for bathing.

### Randomization, allocation concealment, and blinding

Randomization was performed through cluster randomization with crossover, where the unit of randomization was the ICU. Randomization lists were generated by an independent statistician using R software.^( 30 )^ Randomization was performed by the coordinating center using a central online randomization system (RedCap^®^) on the day of each center’s activation. The intervention sequence was disclosed to the investigators only after all the center’s information was recorded in the online system.

This study is an open-label trial in which patients and investigators are not blinded to the study interventions. The visual aspect and smell of the 2% CHG solution prevents the blinding of patients and study personnel.

### Study outcomes

The primary outcome is the incidence of device-associated HCAIs (CLABSI, VAP, and CAUTI) during the ICU stay within each intervention period. The diagnosis of device-associated HCAIs is made according to the *Agência Nacional de Vigilância Sanitária* (ANVISA) definitions.^( 31 )^

The secondary outcomes are the incidence of each specific HCAI (CLABSI, VAP, and CAUTI) within each intervention period, rates of positive microbiological cultures for multidrug-resistant (MDR) pathogens within each intervention period, antibiotic use (measured by days of therapy [DOT] or defined daily dose [DDD]) within each intervention period, ICU and hospital length of stay up to 90 days, and ICU and hospital mortality up to 90 days.

Detailed information on the outcomes and study definitions is available in the Supplementary Material .

### Data collection, data sources, and monitoring

Unidentified patient data are routinely collected for the IMPACTO-MR platform by all ICUs and include data on demographics, baseline characteristics, admission diagnosis, severity score (Simplified Acute Physiology Score [SAPS] 3), ICU and hospital length of stay, and mortality. Additional data on HCAIs are collected through a digital online case report form developed by the HCor data management team using REDCap^®^.^( 32 , 33 )^ Additionally, data on antibiotic use during the ICU stay and the results of microbiological cultures collected during the ICU stay are collected from all participating centers using prescription, pharmacy, and microbiology laboratory records.

Access to all data platforms is password protected and supported by the HCor data management team. Data monitoring is performed by the HCor data management team. All data on HCAIs will be monitored, either *in loco* or online.

### Adverse events

This trial includes only critically ill patients, with an inherent high risk of death and other abnormal findings associated with the underlying illness severity. We will not consider these findings as adverse events unless the investigator suspects or confirms that these events are related to the study interventions.^( 34 )^ All participating centers were trained and encouraged to report all adverse events that were judged to be potentially causally related to the study intervention.

### Sample size

Initially, the study was expected to include between 30 and 50 ICUs of the IMPACTO-MR platform, with an expected sample size of 30,000 patients ( Figure 1S - Supplementary Material ). However, owing to feasibility issues and restrictions on the study’s budget provided by the Brazilian Ministry of Health after December 31, 2023, the sample size was recalculated considering possible variations in the number of clusters, cluster size, and data from the IMPACTO-MR database to estimate the baseline rate of HCAIs. Therefore, by including 12,600 patients (21 clusters with a mean of 600 patients each) with a baseline rate of 6 HCAIs per 1,000 patient-days and a between-cluster variance of 0.6, our study will be able to detect a 9% (relative risk of 0.91) reduction in the incidence of HCAIs, with 80% power and an alpha of 5% ( Figure 2S - Supplementary Material ). Details are provided in the Supplementary Material .

### Statistical analysis

The main analysis will follow the intention-to-treat framework with patient-level data. Each patient will be analyzed according to the group (CHG or soap and water bath) assigned to the ICU at admission, irrespective of the length of stay and the number of days the patient received each intervention. Patients whose stay crosses into another treatment period will be analyzed according to their initial assignment, and patients admitted during the washout period will not be included in the intention-to-treat analysis. We will perform a sensitivity analysis including only patients admitted and discharged during the same intervention period.

The primary outcome of HCAI incidence per 1,000 patient-days will be analyzed using a Poisson generalized mixed linear model with allocation group, study period, and the interaction of allocation group and study period as fixed effects and center as a random effect. Other possible distributions and models might be considered according to the variable’s behavior. We will use the same model to evaluate the incidence per 1000 patient-days of each specific HCAI (CLABSI, VAP, and CAUTI), rates of positive microbiological cultures for multidrug-resistant pathogens, and antibiotic use. Mortality will be assessed using survival and logistic regression models, taking the center, allocation group, and study period as random effects and the interaction of the allocation group and study period as a fixed effect. Generalized mixed linear models with appropriate distributions will be utilized for other outcomes. Preplanned subgroup analyses will be conducted according to the following characteristics: surgical *versus* nonsurgical admission, SAPS 3 score ≤ 40 *versus* > 40, public *versus* private ICU, and ICU length of stay ≤ 7 *versus* > 7 days.

No adjustments for multiplicity will be performed, and a 2-sided p value of less than 0.05 will be considered statistically significant. All the statistical analyses will be performed using R software (R Foundation for Statistical Computing, Vienna, Austria; https://www.R-project.org/).^( 30 )^

### Ethics and dissemination

The trial was designed according to the guidelines for good clinical practice and follows the principles of the Declaration of Helsinki. This study was approved by the HCor Ethical Committee (51470721.6.1001.0060) and by the research ethical committees of all the participant sites.

Given the nature of the study and the minimal risk associated with the intervention, a waiver of consent was requested and approved in all centers.

Confidentiality will be ensured throughout the process, from data collection to data management and the sharing of unidentifiable data when made available. All data generated from this trial will be made available to the trial’s steering committee with a scientifically sound research proposal upon request. All the results will be published in a peer-reviewed medical journal, irrespective of their findings. The steering committee members will author the main paper, along with the principal investigators of each site, respecting the International Committee of Medical Journal Editors (ICMJE) criteria.

## DISCUSSION

The CLEAN-IT trial is the first multicenter, randomized clinical trial evaluating the effect of daily bathing with CHG to reduce HCAIs in critically ill patients in an LMIC; it is also expected to be the largest trial in the literature on the subject.

When designing the CLEAN-IT trial, the steering committee faced important issues that should be discussed. One of the distinct aspects of the study is that we chose to use a 2% CHG solution instead of CHG-impregnated bathing cloths. In Brazil and other LMICs, the financial costs of CHG-impregnated bathing cloths would jeopardize the implementation of the intervention if it proves to be effective on a large scale. Therefore, our aim with this protocol was to evaluate a cheaper and readily available intervention with high external validity.

Additionally, we chose to use patient-days as the denominator for the reporting of HCAI incidence instead of device-days given the simplicity and accuracy of patient-day data collection and the equivalence of both metrics and their use in similar studies.^( 1 , 22 , 35 )^

Another design issue is the rationale for performing a cluster randomized trial instead of an individual randomized trial. Bathing practices in intensive care units are usually interventions decided at the unit level and not at the individual level, allowing more uniform delivery of the intervention, due to both the type of intervention and logistical convenience and acceptability. Furthermore, an intervention like the one being studied could have spillover effects on the microbiology of the intensive care unit that could hamper the identification of the effects of the intervention.

Additionally, given restrictions on the study’s budget provided by the Brazilian Ministry of Health after December 31, 2023, we could not wait for all sites to be approved to initiate the study; therefore, the trial started once each center was approved. Coupled with an initial estimate of 50 centers, this staggered inclusion might lead to transitory imbalances in the recruitment rate of each group. However, we will address this in the analysis by considering the time period of each intervention. This time constraint on the trial also led to the choice of a one-month washout period. While more extended washout periods would be ideal to avoid cross-contamination of ICU-level microbiological effects of the intervention, the trial would be unable to be conducted within the planned time frame.

Although CHG bathing might be a standard practice in some countries, its use is still very limited in Brazil, and its efficacy has yet to be tested in a trial with higher rates of HCAIs and in an LMIC. The CLEAN-IT trial is evaluating a simple and affordable intervention that can help guide future efforts in preventing HCAIs worldwide.

### Perspective

Inclusion of patients in the CLEAN-IT trial ended on December 31, 2023. As we write the draft for this trial protocol, each center is finalizing data collection, and we expect the database to be closed for analysis in late March. A total of 23 centers participated in the CLEAN-IT trial. We expect to perform data analysis and have the results published by the second semester of 2024.

## CONCLUSION

The CLEAN-IT trial involves efforts to study feasible and affordable interventions that might reduce the burden of health care-associated infections in critically ill patients in low- and middle-income countries.

## SUPPLEMENTARY MATERIAL


