# Supplementary material for: Daily Chlorhexidine Bath for Health Care Associated Infection Prevention (CLEAN-IT): protocol for a multicenter cluster randomized crossover open-label trial
Source: Crit Care Sci. 2024 Sep 18;36:e20240053en. doi: 10.62675/2965-2774.20240053-en (PMC11463980; doi:10.62675/2965-2774.20240053-en)
Supplement: Supplementary file 1 [file 2965-2774-ccsci-36-e20240053en-suppl01.pdf]

*Critical Care Science*
https://doi.org/10.62675/2965-2774.20240053-en

SUPPLEMENTARY MATERIAL

# Daily Chlorhexidine Bath for Health Care Associated Infection Prevention (CLEAN-IT): protocol for a multicenter cluster randomized crossover open-label trial

Bruno Martins Tomazini[1,2,3], Thabata Silva Veiga[1], Renato Hideo Nakagawa Santos[1], Viviane Bezerra Campos[1], Samira Martins Tokunaga[1], Elton Sousa Santos[1], Leticia Galvão Barbante[1], Renato da Costa Maia[1], Karina Leal Negrelli[1], Nanci Valeis[1], Eliana Vieira Santucci[1], Ligia Nasi Laranjeira[1], Fernando Azevedo Medrado Jr.[1], Thiago Costa Lisboa[2,4], Bruno Adler Maccagnan Pinheiro Besen[2,3,5], Antônio Paulo Nassar Junior[2,6,7], Viviane Cordeiro Veiga[2,8], Adriano Jose Pereira[2,6], Alexandre Biasi Cavalcanti[1,2] for the IMPACTO-MR Investigators

## SUMMARY

Health-care-associated infections definitions ........ 2
Multidrug resistant microorganism definitions ........ 2
Antibiotic use definition ........ 3
Sample size and power ........ 3
References ........ 4



This is an open access article under the CC BY license (https://creativecommons.org/licenses/by/4.0/).

## HEALTH-CARE-ASSOCIATED INFECTIONS DEFINITIONS

Our study focuses on device associated health-care-associated infections (HCAIs) (central line associated bloodstream infection [CLASBI], catheter associated urinary tract infection [CAUTI], and ventilator associated pneumonia [VAP]), which follow the *Agência Nacional de Vigilância Sanitária* (ANVISA) definitions.[1]

## VENTILATOR ASSOCIATED PNEUMONIA

In this trial, we will use both the clinical and microbiological definitions of VAP according to the following criteria:

### Clinical VAP:

1. Patient under mechanical ventilation for at least 48 hours or mechanical ventilation has been removed in the last day, **AND**
2. New, persistent, or progressive chest imaging infiltrate, opacity or cavitation, **AND**
3. At least one of the signs of increased body temperature > 38.0°C **OR** leucocyte count > 12,000/mL or < 4,000/mL, **AND**
4. Presence of at least one of:
    a. New onset of purulent tracheal secretions, or change in the secretion characteristics, or increase in the amount of respiratory secretions, or increased need of aspiration, **OR**
    b. Worsening of gas exchange, **OR**
    c. Ronchi or crepitation in the lung auscultation, **OR**
    d. Worsening of cough, dyspnea, or tachypnea.

### Microbiological confirmed VAP:

1. Fulfillment of all clinical VAP criteria, **AND**
2. At least one of the results below:
    a. Positive blood cultures, without other infectious source, **OR**
    b. Positive pleural fluid cultures, **OR**
    c. Positive quantitative culture of a lower respiratory tract specimen obtained with a minimal potential for contamination (bronchoalveolar lavage [BAL], protected brush or endotracheal aspirate), **OR**
    d. On BAL bacterioscopy, finding of ≥ 5% of leucocytes and macrophages with microorganisms, **OR**
    e. Positive culture from lung tissue, **OR**
    f. Histopathological exam with evidence of pneumonia, **OR**
    g. Viruses, *Bordetella*, *Legionella*, *Chlamydophila* or *Mycoplasma* identified from a culture of a lower respiratory tract specimen or lung tissue or identified through a microbiological test done for diagnostic or treatment reasons, **OR**
    h. 4-fold increase in IgG values serology for a pathogen (e.g. *influenza*, *Chlamydophila*), **OR**
    i. 4-fold increase in IgG values serology for *Legionella pneumophila* serogroup I titrated to ≥ 1:128 in the acute phase or convalescence by indirect immunofluorescence assay, **OR**
    j. Urinary antigen detection of *Legionella pneumophila* serogroup I.

## CATHETER ASSOCIATED URINARY TRACT INFECTION

1. Patient with urinary catheter for at least 48 hours or urinary catheter has been removed in the last day, **AND**
2. Presence of at least one of:
    a. Body temperature >38.0°C, **OR**
    b. Suprapubic pain or discomfort, **OR**
    c. Lumbar pain or discomfort, **OR**
    d. Dysuria, polyuria, or pollakiuria (only for patients which the urinary catheter has been removed in the last day). **AND**
    e. Positive microbiological culture with $\geq 10^5$ CFU.

## CENTRAL LINE ASSOCIATED BLOODSTREAM INFECTION

1. Patient with central line for at least 48 hours or central line has been removed in the last day, **AND**
2. Pathogenic agent identified in one more hemocultures, **AND**
3. The identified microorganism is not associated with other infection.

## MULTIDRUG RESISTANT MICROORGANISM DEFINITIONS

The operational definition of multidrug resistant microorganisms is described below:

| Microorganism | Resistance profile |
|---|---|
| *Acinetobacter baumannii* | Resistant to carbapenems and/or polymyxins |
| *Pseudomonas aeruginosa* | Resistant to carbapenems and/or polymyxins |
| *Enterobacteriaceae* | Resistant to carbapenems and/or polymyxins (for Enterobacteriaceae naturally sensitive to polymyxins) |
| *Enterococcus faecium* | Resistant to vancomycin |
| *Staphylococcus aureus* | Resistant to methicillin/oxacillin |
| *Coagulase-negative Staphylococcus* | Resistant to methicillin/oxacillin |

## ANTIBIOTIC USE DEFINITION

We will measure antibiotic consumption using the days of therapy (DOT) or defined daily dose (DDD).[2]

The number of days a patient receives an antimicrobial agent (regardless of dose) equals the DOT. Any dose of an antimicrobial received during a 24-hour period represents 1 DOT. For example, in a treatment with a combined regimen of three antimicrobials for 10 days, 30 DOTs would be counted, 10 DOTs for each antimicrobial. For the same clinical indication, if monotherapy was adopted (only 1 antimicrobial), the sum would be 10 DOTs.

The defined daily dose (DDD) is the average maintenance dose per day for a drug used as a main indication in adults. The DDD is calculated by dividing each antibiotic quantity dispensed (in grams) by the World Health Organization average maintenance dose per day for a drug used as a main indication in adults.

## SAMPLE SIZE AND POWER

When the trial was first designed, it was expected to include between 30 and 50 intensive care units (ICUs) of the IMPACTO-MR platform with expected sample size of 30.000 patients (Figure 1S), considering a baseline HCAIs incidence of 10/1,000 patients-day, and between-cluster variation of 0.6. We invited all participating ICUs in the IMPACTO-MR platform to participate in the trial, however, some of the invited ICUs already performed daily chlorhexidine digluconate bathing and were not willing to change their practice, and some centers declined participation due to other institutional reasons. Also, there were restrictions to use the study's budget provided by the Brazilian Ministry of health after December 31st 2023.

After this initial phase of declines, we reviewed our sample size and power estimates, and give the amount of uncertainty in its calculations (effect size, numbers of clusters and clusters sizes) we performed a reviewed sample size and power calculations accounting for these uncertainties. This allowed us for estimate a range of sample size and possible effect sizes reductions for a power between 80% and 90% (Figure 2S).

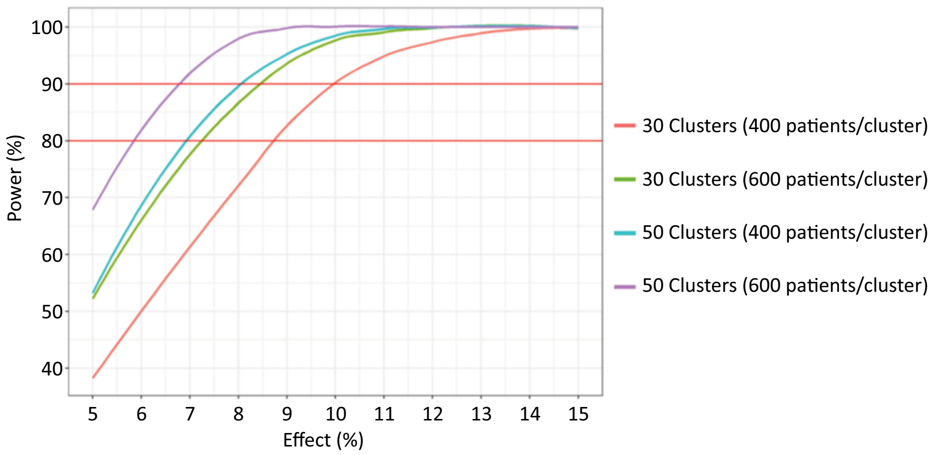


**Figure 1S -** Initial sample size and power calculations.

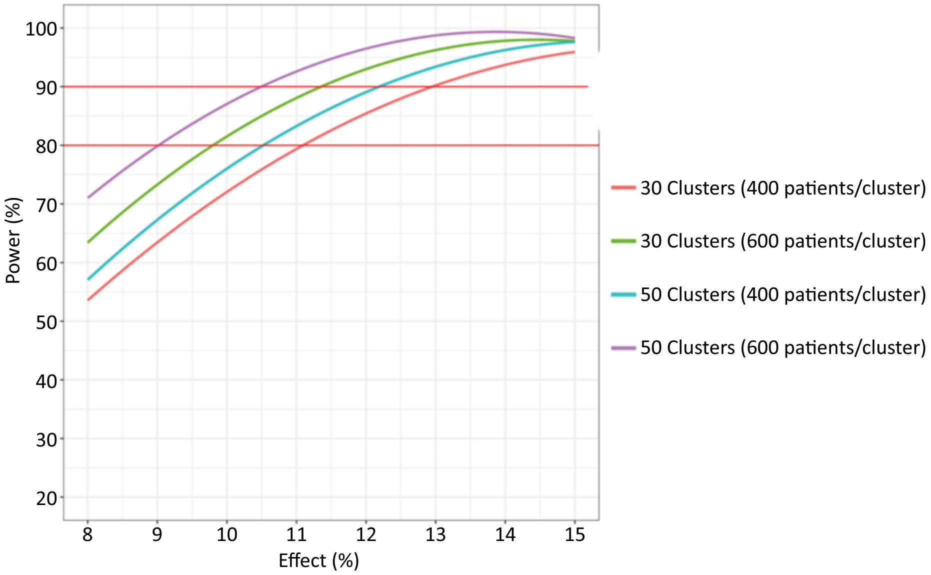


**Figure 2S -** Final sample size and power calculations.

Therefore, we expect to include between 12,600 (21 clusters with mean of 600 patients each) and 19,200 patients (24 clusters with mean of 800 patients each). Considering a baseline rate of 6 HCAIs per 1,000 patients-day, and intracluster correlation coefficient of 0.6, our study will be able to detect a reduction between 9% (relative risk of 0.91) and 11.1% (relative risk of 0.89) with 80% power or a reduction between 10.5% (relative risk of 0.895) and 13% (relative risk of 0.87) with 90% power, with alpha of 5%.

## REFERENCES

1. Brasil. Agência Nacional de Vigilância Sanitária (ANVISA). Critérios diagnósticos de Infecções Relacionadas à Assistência à Saúde. Brasília (DF): ANVISA; 2021.
2. Stanić Benić M, Milanič R, Monnier AA, Gyssens IC, Adriaenssens N, Versporten A, Zanichelli V, Le Maréchal M, Huttner B, Tebano G, Hulscher ME, Pulcini C, Schouten J, Vlahovic-Palcevski V; DRIVE-AB WP1 group. Metrics for quantifying antibiotic use in the hospital setting: results from a systematic review and international multidisciplinary consensus procedure. J Antimicrob Chemother. 2018;73(Suppl 6):vi50-8.
