# Supplementary material for: Daily Chlorhexidine Bath for Health Care Associated Infection Prevention (CLEAN-IT): protocol for a multicenter cluster randomized crossover open-label trial
Source: Crit Care Sci. 2024 Sep 18;36:e20240053en. doi: 10.62675/2965-2774.20240053-en (PMC11463980; doi:10.62675/2965-2774.20240053-en)
Supplement: Translation

# Translation: *Daily Chlorhexidine Bath for Health Care Associated Infection Prevention* (CLEAN-IT): protocolo de um estudo multicêntrico, aberto, randomizado, em cluster com cruzamentos

**DOI:** 10.62675/2965-2774.20240053-pt

**Authors:** Bruno Martins Tomazini, Thabata Silva Veiga, Renato Hideo Nakagawa Santos, Viviane Bezerra Campos, Samira Martins Tokunaga, Elton Sousa Santos, Leticia Galvão Barbante, Renato da Costa Maia, Karina Leal Negrelli, Nanci Valeis, Eliana Vieira Santucci, Ligia Nasi Laranjeira, Fernando Azevedo Medrado, Thiago Costa Lisboa, Bruno Adler Maccagnan Pinheiro Besen, Antônio Paulo Nassar, Viviane Cordeiro Veiga, Adriano José Pereira, Alexandre Biasi Cavalcanti

**Affiliations:** 1 Brasil Instituto de Pesquisa HCor, HCor-Hospital do Coração - São Paulo (SP), Brasil.; 2 Brasil Brazilian Research in Intensive Care Network (BRICNet) - São Paulo (SP), Brasil.; 3 Brasil Hospital Sírio-Libanês - São Paulo (SP), Brasil.; 4 Brasil Hospital de Clínicas de Porto Alegre, Universidade Federal do Rio Grande do Sul -Porto Alegre (RS), Brasil.; 5 Brasil Unidade de Terapia Intensiva Médica, Hospital das Clínicas, Faculdade de Medicina, Universidade de São Paulo - São Paulo (SP), Brasil.; 6 Brasil Hospital Israelita Albert Einstein - São Paulo (SP), Brasil.; 7 Brasil AC Camargo Cancer Center - São Paulo (SP), Brasil.; 8 Brasil BP - A Beneficência Portuguesa de São Paulo - São Paulo (SP), Brasil.

**Keywords:** Infecção hospitalar, Clorexidina, Anti-infecciosos, tópico, Banhos, Sabões, Doença crítica, Infecções nosocomiais, Sepse, Tempo de permanência, Unidades de terapia intensiva

## Abstract

**Fundamentos:**

Pacientes críticos têm maior risco de infecções relacionadas à assistência à saúde devido ao uso de vários dispositivos (infecção da corrente sanguínea associada a cateter central, infecção do trato urinário associada ao cateter e pneumonia associada à ventilação mecânica), o que representa uma ameaça significativa para essa população. Entre as várias estratégias, o banho diário com digluconato de clorexidina, um antisséptico solúvel em água, foi estudado como intervenção para diminuir a incidência de infecções relacionadas à assistência à saúde na unidade de terapia intensiva; no entanto, sua capacidade de reduzir todas as infecções relacionadas à assistência à saúde devido a vários dispositivos não está clara. Desenhamos o estudo *Daily Chlorhexidine Bath for Health Care Associated Infection Prevention* (CLEAN-IT) para avaliar se o banho diário com digluconato de clorexidina reduz a incidência de infecções relacionadas à assistência à saúde em pacientes críticos em comparação com o banho de água e sabão.

**Métodos:**

O estudo CLEAN-IT é um estudo clínico multicêntrico, aberto, randomizado e em *cluster* com cruzamentos. Todos os pacientes adultos admitidos nas unidades de terapia intensiva participantes serão incluídos no estudo. Cada grupo (unidade de terapia intensiva) será randomizado para realizar o banho inicial com digluconato de clorexidina ou banho de água e sabão com cruzamento por um período de 3 a 6 meses, dependendo do momento de entrada de cada centro no estudo, com um período de *washout* de 1 mês entre os banhos com digluconato de clorexidina e as transições para os banhos de água e sabão. O desfecho primário é a incidência de infecções relacionadas à assistência à saúde devido a dispositivos. Os desfechos secundários são a incidência de cada infecção específica relacionada à assistência à saúde, as taxas de coleções microbiológicas positivas para microrganismos multirresistentes, o uso de antibióticos, o tempo de internação na unidade de terapia intensiva e no hospital e a mortalidade na unidade de terapia intensiva e no hospital.

**Conclusão:**

O estudo CLEAN-IT será usado para estudar intervenções viáveis e acessíveis que possam reduzir a incidência de infecções relacionadas à assistência à saúde em pacientes em estado crítico.

## INTRODUÇÃO

As infecções relacionadas à assistência à saúde (IRAS) são doenças evitáveis comuns em pacientes críticos hospitalizados^( 1 )^e estão associadas ao aumento dos custos da assistência à saúde, ao tempo de internação e à mortalidade.^( 2 - 5 )^Os pacientes críticos correm um risco maior de contrair IRAS devido a uma infinidade de fatores, como o uso de dispositivos invasivos (tubos endotraqueais, cateteres centrais e cateteres urinários) e sua doença crítica subjacente. Práticas como assistência, pacotes de inserção de dispositivos e higiene das mãos são os pilares da prevenção de infecções^( 6 - 9 )^e um dos principais focos dos serviços de controle de infecções e prevenção, com vários ensaios clínicos avaliando estratégias de prevenção de infecções no ambiente de terapia intensiva.^( 10 - 15 )^

O digluconato de clorexidina (CHG - *chlorhexidine digluconate* ) é um agente antisséptico tópico, solúvel em água e com baixa formação de espuma, incluído na lista de medicamentos essenciais da Organização Mundial da Saúde.^( 16 )^O CHG apresenta atividade contra bactérias *Gram* -positivas e *Gram* -negativas, leveduras e vírus lipofílicos, com possível benefício de atividade antibacteriana residual.^( 17 )^O CHG tem sido usado em curativos de cateteres vasculares impregnados, preparação da pele para procedimentos invasivos, banhos pré-operatórios e descontaminação oral.^( 18 - 20 )^

O uso de banhos diários com CHG para reduzir as IRAS em pacientes críticos foi avaliado em vários estudos, com resultados mistos que indicam redução do risco de algumas infecções (infecção da corrente sanguínea associada a cateter central [ICSAC] e bacteremia) e aumento do risco de outras (pneumonia associada à ventilação mecânica [PAV]).^( 14 , 15 , 21 , 22 )^O papel do banho de CHG na redução das IRAS não foi adequadamente avaliado em países de baixa e média renda (PBMRs), onde a incidência e a carga das IRAS são maiores.^( 1 , 23 , 24 )^É desejável um desenho randomizado em grupos ao estudar intervenções para reduzir as IRAS, dado o possível benefício do efeito de transbordamento e a redução da contaminação entre os grupos tratamento e controle.^( 25 , 26 )^

O *Daily Chlorhexidine Bath for Health Care Associated Infection Prevention* (CLEAN-IT) é um estudo randomizado em cluster com cruzamentos para avaliar o uso de banhos diários com CHG em pacientes críticos na redução de IRAS associadas a dispositivos (ICSAC, infecção do trato urinário associada a cateter [ITUAC] e PAV) em comparação com banhos de água e sabão.

## MÉTODOS

Neste relato, seguiram-se as diretrizes do *Standard Protocol Items: Recommendations for Interventional Trials* (SPIRIT).^( 27 )^O relato final do estudo seguirá a declaração *Consolidated Standards of Reporting Trials* (CONSORT) e sua extensão para estudos randomizados em grupos.^( 28 )^O estudo foi registrado no ClinicalTrials.gov (NCT05485051) antes da inclusão do primeiro paciente.

### Desenho do estudo

O estudo CLEAN-IT é um ensaio clínico multicêntrico, aberto, randomizado em *cluster* com cruzamentos iniciado por um investigador, que avalia a eficácia dos banhos diários com CHG na redução da incidência de IRAS associadas a dispositivos em pacientes críticos em comparação com banhos de água e sabão.

O estudo foi realizado em 23 unidades de terapia intensiva (UTIs) brasileiras e está vinculado à IMPACTO-MR,^( 29 )^uma plataforma de pesquisa que coleta dados observacionais prospectivos de mais de 50 UTIs brasileiras.

Cada UTI participante é um grupo randomizado para realizar as intervenções (banhos de CHG ou banhos de água e sabão) com cruzamento por um período de 3 a 6 meses, dependendo do momento da entrada de cada centro no estudo, com um período de *washout* de 1 mês entre as transições de banhos de CHG e banhos de água e sabão. A figura 1 mostra todas as sequências de alocação possíveis para as UTIs participantes.

**Figura 1:** Esquema das possíveis sequências de alocação.

CHG - digluconato de clorexidina.

### População e intervenções do estudo

Incluíram-se no estudo todos os pacientes adultos ≥ 18 anos de idade internados nas UTIs participantes. Os pacientes foram excluídos se alérgicos a CHG.

### Banhos de digluconato de clorexidina

Todos os pacientes na UTI durante o período de banhos de CHG que não atenderam aos critérios de exclusão foram banhados diariamente com CHG a 2%. Todos os centros participantes receberam CHG a 2% e luvas (luvas macias de poliéster específicas para banho hospitalar) para todos os períodos de banhos de CHG.

O procedimento de banho consistia na aplicação de CHG a 2% (não era permitida nenhuma forma de diluição da solução de CHG a 2%) com as luvas fornecidas e no uso da solução para banhar o paciente, garantindo que todas as superfícies de banho aplicáveis fossem cobertas com CHG a 2% e que não houvesse áreas com sujeira visível no paciente. As superfícies de banho aplicáveis incluíam todas as superfícies do corpo, exceto os olhos, o ouvido interno, a boca e as áreas com perda de continuidade da pele (por exemplo: feridas abertas e regiões queimadas). O banho era realizado no sentido craniocaudal (da cabeça aos pés), sendo que a última área banhada era a região genital, que era obrigatoriamente lavada com um novo par de luvas. Os pacientes foram enxaguados de acordo com as práticas usuais de cada centro.

Em casos de sujeira extensa, a limpeza da área suja poderia ser realizada antes do banho de CHG a 2%. Outros aspectos relacionados ao banho (por exemplo: hora do dia, número de funcionários envolvidos em cada banho e banho à beira do leito ou no chuveiro) e práticas e pacotes de controle de infecção seguiram as práticas usuais de cada centro.

### Banhos de água e sabão

Todos os pacientes na UTI durante o período de banhos de água e sabão e os períodos de *washout* foram banhados diariamente segundo as práticas usuais de cada centro. Proibiu-se o CHG para banho em qualquer concentração durante esses períodos. A única exceção foi o banho pré-operatório para pacientes cirúrgicos, que foi permitido nos centros com essa prática. Outros aspectos relacionados ao banho (por exemplo: hora do dia, número de funcionários envolvidos em cada banho e banho à beira do leito ou no chuveiro) e práticas e pacotes de controle de infecção seguiram as práticas usuais de cada centro.

### Período de washout

O período de *washout* foi definido como 1 mês entre os diferentes períodos de intervenção (período de banhos de CHG e período de banhos de água e sabão). Durante esse período, todos os centros usaram água e sabão para o banho.

### Randomização, ocultação de alocação e cegamento

A randomização foi realizada por meio de randomização em grupos com cruzamento, em que a unidade de randomização foi a UTI. As listas de randomização foram geradas por um estatístico independente, usando o programa R.^( 30 )^A randomização foi realizada pelo centro de coordenação usando um sistema central de randomização online (RedCap^®^) no dia da ativação de cada centro. A sequência da intervenção foi divulgada aos pesquisadores somente depois que todas as informações do centro foram registradas no sistema *on-line* .

Trata-se de um estudo aberto no qual os pacientes e os pesquisadores não são cegados para as intervenções do estudo. O aspecto visual e o cheiro da solução de CHG a 2% impedem o cegamento dos pacientes e da equipe do estudo.

### Desfechos do estudo

O desfecho primário é a incidência de IRAS associadas a dispositivos (ICSAC, PAV e ITUAC) durante a internação na UTI em cada período de intervenção. O diagnóstico de IRAS associadas a dispositivos é feito conforme as definições da Agência Nacional de Vigilância Sanitária (Anvisa).^( 31 )^

Os desfechos secundários são a incidência de cada IRAS específica (ICSAC, PAV e ITUAC) em cada período de intervenção, taxas de coleções microbiológicas positivas para microrganismos multirresistentes (MMRs) em cada período de intervenção, uso de antibióticos (medido por dias de terapia [DOT - *days of therapy* ] ou dose diária definida [DDD]) em cada período de intervenção, tempo de internação na UTI e no hospital até 90 dias e mortalidade na UTI e no hospital até 90 dias.

Informações detalhadas sobre os desfechos e as definições do estudo estão disponíveis no Material Suplementar.

### Coleta de dados, fontes de dados e monitoramento

Os dados de pacientes desidentificados são sistematicamente registrados na plataforma IMPACTO-MR por todas as UTIs e incluem dados demográficos, características basais, diagnóstico de admissão, pontuação de gravidade ( *Simplified Acute Physiology Score* [SAPS] 3), tempo de internação na UTI e no hospital e mortalidade. Outros dados sobre IRAS são coletados por meio de um formulário digital *on-line* de relato de caso desenvolvido pela equipe de tratamento de dados do HCor usando o REDCap^( 32 , 33 )^Além disso, os dados de uso de antibióticos durante a internação na UTI e os resultados das coleções microbiológicas coletadas durante a internação na UTI são coletados de todos os centros participantes por meio de registros de prescrição, farmácia e laboratório de microbiologia.

O acesso a todas as plataformas de dados é protegido por senha e assistido pela equipe de tratamento de dados do HCor. O monitoramento de dados é realizado pela equipe de tratamento de dados do HCor. Todos os dados sobre IRAS serão monitorados, *in loco* ou *on-line* .

### Eventos adversos

Este estudo inclui apenas pacientes críticos, com alto risco inerente de morte e outros achados anormais associados à gravidade da doença subjacente. Não consideraremos esses achados como eventos adversos, a menos que o investigador suspeite ou confirme que esses eventos estão relacionados às intervenções do estudo.^( 34 )^Todos os centros participantes foram treinados e incentivados a relatar todos os eventos adversos que foram julgados como potencialmente relacionados à intervenção do estudo.

### Tamanho da amostra

Inicialmente, esperava-se que o estudo incluísse entre 30 e 50 UTIs da plataforma IMPACTO-MR, com tamanho de amostra esperado de 30 mil pacientes (Figura 1S - Material Suplementar). No entanto, devido a problemas de viabilidade e restrições no orçamento do estudo, fornecido pelo Ministério da Saúde, após 31 de dezembro de 2023, o tamanho da amostra foi recalculado, considerando possíveis variações no número de grupos, tamanho dos grupos e dados do banco de dados IMPACTO-MR para estimar a taxa basal de IRAS. Portanto, ao incluir 12.600 pacientes (21 grupos com uma média de 600 pacientes cada) com taxa basal de 6 IRAS por mil pacientes-dia e variação entre grupos de 0,6, nosso estudo é capaz de detectar uma redução de 9% (risco relativo de 0,91) na incidência de IRAS, com 80% de poder e um alfa de 5% (Figura 2S - Material suplementar). Os detalhes encontram-se no Material Suplementar.

### Análise estatística

A análise principal seguirá a estrutura de intenção de tratar com dados no nível do paciente. Cada paciente será analisado conforme o grupo (CHG ou banho de água e sabão) atribuído à UTI na admissão, independentemente do tempo de internação e do número de dias em que o paciente recebeu cada intervenção. Os pacientes cuja internação coincidir com outro período de tratamento serão analisados conforme sua atribuição inicial, e os pacientes admitidos durante o período de *washout* não serão incluídos na análise de intenção de tratar. Realizaremos uma análise de sensibilidade incluindo apenas os pacientes admitidos e que receberam alta durante o mesmo período de intervenção.

O desfecho primário da incidência de IRAS por mil pacientes-dia será analisado usando um modelo linear misto generalizado de Poisson com grupo de alocação, o período de estudo e a interação entre grupo de alocação e período de estudo, como efeitos fixos, e o centro como efeito aleatório. Outras distribuições e modelos possíveis podem ser considerados conforme o comportamento da variável. O mesmo modelo será usado para avaliar a incidência por mil pacientes-dia de cada IRAS específica (ICSAC, PAV e ITUAC), taxas de coleções microbiológicas positivas para microrganismos multirresistentes e uso de antibióticos. A mortalidade será avaliada por meio de modelos de sobrevida e regressão logística, considerando o centro, o grupo de alocação e o período do estudo como efeitos aleatórios e a interação entre grupo de alocação e período do estudo como um efeito fixo. Modelos lineares mistos generalizados com distribuições apropriadas serão utilizados para outros resultados. Análises de subgrupo pré-planejadas serão conduzidas de acordo com as seguintes características: admissão cirúrgica *versus* não cirúrgica, SAPS 3 ≤ 40 *versus* > 40, UTI pública *versus* privada e tempo de internação na UTI ≤ 7 *versus* > 7 dias.

Não serão realizados ajustes de multiplicidade e será considerado estatisticamente significativo valor de p bilateral inferior a 0,05. Todas as análises estatísticas serão realizadas com o programa R (R Foundation for Statistical Computing, Viena, Áustria; https://www.R-project.org/).^( 30 )^

### Ética e divulgação

O estudo foi elaborado segundo as diretrizes de boas práticas clínicas e segue os princípios da Declaração de Helsinque. Este estudo foi aprovado pelo Comitê de Ética do HCor (51470721.6.1001.0060) e pelos Comitês de Ética em Pesquisa de todos os centros participantes.

Dada a natureza do estudo e o risco mínimo associado à intervenção, solicitou-se e aprovou-se a dispensa de consentimento em todos os centros.

A confidencialidade será garantida durante todo o processo, desde a coleta até o tratamento dos dados, além do compartilhamento de dados não identificáveis, quando disponibilizados. Todos os dados gerados por este estudo serão disponibilizados ao comitê diretor do estudo com uma proposta de pesquisa cientificamente sólida, mediante solicitação. Todos os resultados serão publicados em uma revista médica revisada por pares, independentemente de suas conclusões. Os membros do comitê diretor serão os autores do artigo principal, com os pesquisadores principais de cada local, respeitando os critérios do *International Committee of Medical Journal Editors* (ICMJE).

## DISCUSSÃO

O estudo CLEAN-IT é o primeiro ensaio clínico multicêntrico e randomizado que avalia o efeito de banhos diários de CHG para reduzir IRAS em pacientes críticos em um país de baixa renda; espera-se também que seja o maior estudo da literatura sobre o assunto.

Ao desenhar o estudo CLEAN-IT, o comitê diretor enfrentou questões importantes que devem ser discutidas. Um dos aspectos distintos do estudo é que optamos por usar uma solução de CHG a 2% em vez de panos de banho impregnados com CHG. No Brasil e em outros PBMRs, os custos financeiros dos panos de banho impregnados com CHG prejudicariam a implementação da intervenção caso ela se mostre eficaz em grande escala. Portanto, nosso objetivo com esse protocolo foi avaliar uma intervenção mais barata e prontamente disponível com alta validade externa.

Além disso, optamos por usar pacientes-dia como denominador para o relato da incidência de IRAS, em vez de dispositivos-dia, dada a simplicidade e a precisão da coleta de dados de pacientes-dia e a equivalência de ambas as métricas e seu uso em estudos semelhantes.^( 1 , 22 , 35 )^

Outra questão do desenho é a justificativa para a realização de um estudo randomizado em grupos, em vez de um estudo randomizado individual. As práticas de banho em UTIs geralmente são intervenções decididas em nível de UTI, não em nível individual, o que permite uma aplicação mais uniforme da intervenção, devido ao tipo de intervenção, à conveniência logística e à aceitabilidade. Além disso, uma intervenção como a que está sendo estudada pode ter efeitos indiretos sobre a microbiologia da UTI, o que pode dificultar a identificação dos efeitos da intervenção.

Além disso, dadas as restrições no orçamento do estudo fornecido pelo Ministério da Saúde após 31 de dezembro de 2023, não podíamos esperar que todos os locais fossem aprovados para iniciar o estudo; portanto, o estudo começou assim que cada centro foi aprovado. Com estimativa inicial de 50 centros, essa inclusão escalonada pode levar a desequilíbrios transitórios na taxa de recrutamento de cada grupo. No entanto, trataremos disso na análise, considerando o período de tempo de cada intervenção. Essa restrição de tempo no estudo também levou à escolha de um período de *washout* de 1 mês. Embora períodos de *washout* mais longos fossem ideais para evitar a contaminação cruzada dos efeitos microbiológicos da intervenção no nível da UTI, o estudo não poderia ser realizado dentro do prazo planejado.

Embora o banho de CHG possa ser uma prática padrão em alguns países, seu uso ainda é muito limitado no Brasil, e sua eficácia ainda não foi testada em um estudo com taxas mais altas de IRAS e em um país de baixa renda. O estudo CLEAN-IT está avaliando uma intervenção simples e acessível, que pode ajudar a orientar futuros esforços na prevenção de IRAS em todo o mundo.

### Perspectiva

A inclusão de pacientes no estudo CLEAN-IT terminou em 31 de dezembro de 2023. Enquanto escrevemos o esboço deste protocolo de estudo, cada centro está finalizando a coleta de dados, e esperamos que o banco de dados seja fechado para análise no fim de março. Ao todo, 23 centros participaram do estudo CLEAN-IT. Esperamos realizar a análise dos dados e publicar os resultados até o segundo semestre de 2024.

## CONCLUSÃO

O estudo CLEAN-IT envolve esforços para estudar intervenções viáveis e acessíveis que possam reduzir o ônus das infecções relacionadas à assistência à saúde em pacientes críticos em países de baixa e média renda.

## MATERIAL SUPLEMENTAR
